# Impact of the COVID-19 pandemic on total, sex- and age-specific all-cause mortality in 20 countries worldwide during 2020: results from the C-MOR project

**DOI:** 10.1093/ije/dyac170

**Published:** 2022-08-27

**Authors:** Christiana A Demetriou, Souzana Achilleos, Annalisa Quattrocchi, John Gabel, Elena Critselis, Constantina Constantinou, Nicoletta Nicolaou, Giuseppe Ambrosio, Catherine M Bennett, Nolwenn Le Meur, Julia A Critchley, Laust Hvas Mortensen, Jose Manuel Rodriguez-Llanes, Mario Chong, Gleb Denissov, Petra Klepac, Lucy P Goldsmith, Antonio José Leal Costa, Terje P Hagen, Marie Chan Sun, Qian Huang, Nataliia Pidmurniak, Inbar Zucker, Joseph Cuthbertson, Bo Burström, Manuel Barron, Ivan Eržen, Fabrizio Stracci, Wilson Calmon, Cyndy Martial, Olesia Verstiuk, Zalman Kaufman, Wenjing Tao, Maia Kereselidze, Nino Chikhladze, Claudia Zimmermann, Eva Schernhammer, Antonis Polemitis, Andreas Charalambous

**Affiliations:** Department of Primary Care and Population Health, University of Nicosia Medical School, Nicosia, Cyprus; Department of Primary Care and Population Health, University of Nicosia Medical School, Nicosia, Cyprus; Department of Primary Care and Population Health, University of Nicosia Medical School, Nicosia, Cyprus; University of Nicosia Medical School, Nicosia, Cyprus; Department of Primary Care and Population Health, University of Nicosia Medical School, Nicosia, Cyprus; Department of Basic and Clinical Sciences, University of Nicosia Medical School, Nicosia, Cyprus; Department of Basic and Clinical Sciences, University of Nicosia Medical School, Nicosia, Cyprus; Department of Medicine, University of Perugia School of Medicine, Perugia, Italy; Institute for Health Transformation, Deakin University, Burwood, Australia; University of Rennes, EHESP, CNRS, Inserm, Arènes—UMR 6051, RSMS—U 1309, Rennes, France; Population Health Research Institute, St George’s, University of London, London, UK; Department of Methods and Analysis, Statistics Denmark, Copenhagen Oe, Denmark; European Commission Joint Research Centre, Ispra, VA, Italy; Departamento de Ingeniería, Universidad del Pacífico, Lima, Peru; Estonian Causes of Death Registry, National institute for Health Development, Tallinn, Estonia; Department of Communicable Diseases, National Institute of Public Health, Ljubljana, Slovenia; Institute for Infection and Immunity, and Population Health Research Institute, St George's, University of London, London, UK; Institute of Studies in Collective Health (IESC), Federal University of Rio de Janeiro, Rio de Janeiro, Brazil; Department of Health Management and Health Economics, Institute of Health and Society, University of Oslo, Oslo, Norway; Department of Medicine, Faculty of Medicine and Health Sciences, University of Mauritius, Réduit, Mauritius; Department of Geography, Center for Rural and Primary Healthcare, University of South Carolina, Columbia, SC, USA; Department of Medicine, Bogomolets National Medical University, Kyiv, Ukraine; School of Public Health, Ministry of Health, Ramat Gan, Israel; Disaster Resilience Initiative, Monash University, Clayton,VIC, Australia; Department of Global Public Health, Karolinska Institutet, Stockholm, Sweden; Department of Economics, Universidad del Pacifico Av Sanchez Cerro, Lima, Peru; School of Public Health, National Institute of Public Health, Medical Faculty, University of Ljubljana, Ljubljana, Slovenia; Public Health Section, Department of Medicine and Surgery, University of Perugia, Piazza Lucio Severi, Perugia, Italy; Institute of Mathematics and Statistics, Fluminense Federal University, Niteroi, Brazil; Department of Demography, Statistics Mauritius, LIC Centre, Port Louis, Mauritius; University of Nicosia Medical School, Nicosia, Cyprus; Israeli Center of Disease Control, Ministry of Health, Ramat Gan, Israel; Department of Global Public Health, Karolinska Institutet, Stockholm, Sweden; Department of Molecular Medicine and SURGERY, Karolinska Institutet, Stockholm, Sweden; National Center for Disease Control and Public Health, Tbilisi, Georgia; Faculty of Medicine, Ivane Javakhishvili Tbilisi State University, Tbilisi, Georgia; Department of Epidemiology, Center for Public Health, Medical University of Vienna, Vienna, Austria; Department of Epidemiology, Center for Public Health, Medical University of Vienna, Vienna, Austria; University of Nicosia, Nicosia, Cyprus; University of Nicosia Medical School, Nicosia, Cyprus

**Keywords:** COVID-19, SARS-CoV-2, all-cause mortality, excess mortality, pandemic, global impact, infection control

## Abstract

**Background:**

To understand the impact of the COVID-19 pandemic on mortality, this study investigates overall, sex- and age-specific excess all-cause mortality in 20 countries, during 2020.

**Methods:**

Total, sex- and age-specific weekly all-cause mortality for 2015–2020 was collected from national vital statistics databases. Excess mortality for 2020 was calculated by comparing weekly 2020 observed mortality against expected mortality, estimated from historical data (2015–2019) accounting for seasonality, long- and short-term trends. Crude and age-standardized rates were analysed for total and sex-specific mortality.

**Results:**

Austria, Brazil, Cyprus, England and Wales, France, Georgia, Israel, Italy, Northern Ireland, Peru, Scotland, Slovenia, Sweden, and the USA displayed substantial excess age-standardized mortality of varying duration during 2020, while Australia, Denmark, Estonia, Mauritius, Norway, and Ukraine did not. In sex-specific analyses, excess mortality was higher in males than females, except for Slovenia (higher in females) and Cyprus (similar in both sexes). Lastly, for most countries substantial excess mortality was only detectable (Austria, Cyprus, Israel, and Slovenia) or was higher (Brazil, England and Wales, France, Georgia, Italy, Northern Ireland, Sweden, Peru and the USA) in the oldest age group investigated. Peru demonstrated substantial excess mortality even in the <45 age group.

**Conclusions:**

This study highlights that excess all-cause mortality during 2020 is context dependent, with specific countries, sex- and age-groups being most affected. As the pandemic continues, tracking excess mortality is important to accurately estimate the true toll of COVID-19, while at the same time investigating the effects of changing contexts, different variants, testing, quarantine, and vaccination strategies.

Key Messages

- This study highlights that, among the investigated countries, the excess mortality burden during the COVID-19 pandemic disproportionally affected specific countries such as Peru, the USA, Slovenia, England and Wales, Brazil, Italy, Northern Ireland and Georgia.
- In sex-specific analyses, excess all-cause mortality was higher in males than in females for all countries, except for Slovenia where excess mortality was higher in females and for Cyprus where excess mortality was similar in both sexes.
- For most countries substantial excess mortality was only detectable or was higher in the oldest age group investigated; Peru showed substantial excess mortality even in younger age groups.
- Further understanding of the determinants of excess mortality is warranted, in order to strengthen health resilience in the countries and population groups impacted the most.

## Introduction

Two years following the first report of cases of a ‘viral pneumonia’ of unknown cause in Wuhan, China, the true toll of the COVID-19 pandemic remains largely underestimated and its determinants are only partially understood.

Nationally published COVID-19 mortality estimates might underestimate or overestimate the actual mortality burden attributed to the pandemic^1^ due to reasons including i) limited testing, which precluded deaths from being categorized as COVID-19 deaths;^2^^,^^3^ ii) deaths attributed as COVID-19 deaths on the basis of someone testing positive, which led to over-counting in some jurisdictions; iii) a delay in the processing of death certificates, which created a lag in data;^4^^,^^5^ and iv) the burden to the healthcare systems and delays in health seeking behaviour due to COVID-19-related anxiety, which exacerbated deaths due to other non-COVID related causes. As an example, an indirect effect of the COVID-19 pandemic was the subversion of emergency room and hospital functioning to cope with the surge of COVID-positive patients, which may have resulted in lack of proper management of other potentially life-threatening conditions.^6^

Still, in certain contexts, undercounting mortality related to COVID-19 could have been rapidly overturned as tests were more readily available and made mandatory for all hospital admissions, increasing the likelihood to misclassify unrelated deaths as COVID-19 deaths, leading to likely overestimation of the true COVID-19 related mortality. As a result, differences in testing and definitions make the reported number of COVID-19 deaths not comparable across populations.^7^

One way to address the aforementioned methodological challenges and obtain more accurate estimates of the toll of the pandemic is to estimate the excess all-cause mortality experienced by countries, by comparing the observed to the expected number of deaths during a specified time window. The expected number of deaths can be predicted using historical data and time series analyses correcting for seasonality and other secular trends.^7^

To date, most studies investigating excess mortality focused on single countries or world regions during the first months of the pandemic and have mostly relied on publicly available data which are often provisional depending on the frequency of updates and the time window between the study period and data extraction.^4^^,^^8–17^ Furthermore, despite differential incidence rates and comorbidities leading to increased probability to adverse outcomes and deaths between age and sex groups, very few studies on more than one country investigated sex and age-specific excess mortality for the whole of 2020.^18^^,^^19^

In an attempt to better understand the impact of the COVID-19 pandemic on mortality, an international consortium, namely the COVID-19 MORtality (C-MOR) Consortium, consisting of over 50 institutions across 52 countries and six continents was formed.^7^ The consortium sought to include countries worldwide without restriction and focused all analyses on data from national primary sources. The present study investigates overall, sex and age-specific excess all-cause mortality in 20 countries, during the whole of 2020.

## Methods

### Data acquisition

Mortality data, collected and provided by partners from 20 countries participating in the international consortium were used in this investigation (including Australia, Austria, Brazil, Cyprus, Denmark, Estonia, France, Georgia, Israel, Italy, Mauritius, Norway, Peru, Slovenia, Sweden, countries of the United Kingdom (UK; England and Wales, Scotland and Northern Ireland), Ukraine and the United States of America (USA)). Total, sex and age-specific weekly all-cause mortality for 2015–2020 was collected from national vital statistics databases, made either publicly available or with restricted access. All countries of the consortium were asked to provide data without a pre-requisite for the civil registration and/or vital statistics system(s) of the country to be of high quality and without a requirement for information to be available in specific age groups. However, collection of weekly mortality estimates was imperative to allow for a more detailed investigation into the timing of excess mortality for each country during 2020. A picture of excess mortality with sufficient granularity provides a more accurate representation of the experience of countries and is of paramount importance for the investigation of drivers and determinants of the excess mortality, such as timing of pandemic waves, seasonality, and government control measures. Depending on the country, all-cause mortality was reported by either ISO week, starting on Monday; Epi week, starting on Sunday; or other national counting week system.

The national primary data sources used in this study and endorsed by the national partners were cross-checked against publicly available data for countries for which information was available. Any minor inconsistencies observed can be explained by retrospective addition of cases and/or delays in reporting of deaths. In addition, internal consistency and quality checks were performed on the data prior to analysis. Data were collected during June and July 2021, several months after the end of the study period, to account for reporting delays (ranging from a few days to a few weeks)^4^^,^^5^ and to allow enough time for data consolidation by reporting authorities toward better data quality.^20^ The national data source and time unit used per country, as well as data availability exceptions for specific population groups, are summarized in Supplementary Table S1 (available as Supplementary data at *IJE* online).

### Statistical analysis

Total and sex-specific excess mortality for 2020 was calculated by comparing weekly 2020 crude mortality rate (CMR) and age-standardized (ASMR) mortality rate (per 100 000 population) against a baseline mortality (expected weekly mortality rate in 2020) estimated based on historical data (2015–2019) accounting for seasonality, and long- and short-term trends.^21–23^ For age-specific excess mortality, only non-standardized mortality rates were used.

For the calculation of mortality rates, total, age and sex-specific mid-year population estimates for the participating countries were obtained from the World bank,^24^ except for the UK nations for which sub-level data from the Office for National Statistics^25^ was used, and for Cyprus for which Eurostat data^26^ was used to include only the population in the Republic of Cyprus government-controlled area.

CMRs were calculated for total population and sex-specific groups using Equation (1) and age-specific mortality rates were calculated using Equation (2) (Supplementary Methods, available as Supplementary data at *IJE* online). Weekly ASMRs were calculated as a weighted average of the age specific mortality rates provided by each country using the WHO World Standard Population 2000–2025^27^ (Equation (3), Supplementary Methods). Because several countries did not report weekly mortality by consistent 5-year or more granular age groups, the method and formula proposed by Klimkin et al. (2021)^28^ was used for the age standardisation. The aggregate age groups created for each country, based on the provided age-specific all-cause mortality data, are shown in Supplementary Table S2 (available as Supplementary data at *IJE* online). This method is not as robust as standardisation using detailed 5-year age groups. However, its results have been shown to only slightly deviate in a downward shift from the ASMR obtained using 5-year age groups, with very close (within 5%) agreement in years closer to 2020.^28^ ASMRs could not be estimated for Scotland due to the lack of age-specific all-cause mortality data (Supplementary Table S1, available as Supplementary data at *IJE* online).

Expected weekly mortality rate for 2020 was modelled using Poisson regression assuming a quasi-Poisson distribution to account for over-dispersion in the weekly mortality rates as described elsewhere.^7^ The residual variation was corrected for skewness by applying a 2/3 power transformation before the computation of the expected 95% confidence intervals.^21^ Standard deviation of the residuals was derived from the expected interval [i.e. (upper expected 95% confidence interval—expected number of deaths)/1.96]. The same model was applied to each country, for total population as well as for sex-specific and age-specific population groups, separately. Age groups <65 *vs.* 65+ years and/or <70 *vs.* 70+ years (depending on available data) were compared, to ensure a sufficient number of deaths in each age group for model robustness. For the countries that showed excess mortality in the younger age group (<65 or <70 years), the analysis was additionally performed for two age subgroups, namely <45 years and 45–64 years, or <50 and 50–69 years.

The regression models were built on complete weeks and any truncated weeks were excluded. Expected mortality rates were estimated for the corresponding complete weeks only. Truncated weeks are usually a result of the different death counts observed around Christmas and New Year,^29^ and these included week 53 (applicable for Australia, Austria, Cyprus, England and Wales, Estonia, France, Georgia, Mauritius, Northern Ireland, Norway, Peru, Scotland, Slovenia, Sweden, and Ukraine), week 52 for England and Wales and Scotland, weeks 51–52 for Northern Ireland, and week 1 for Mauritius. For all countries, observed and expected weekly mortality rates for 2020 were each summed up to week 52, except for England and Wales and Scotland (up to week 51), N. Ireland (up to week 50), and Mauritius (weeks 2–52).

Then, the cumulative expected 2020 mortality rate was subtracted from the cumulative observed 2020 mortality rate to obtain an estimate of excess mortality for the whole of 2020. The statistical significance of excess mortality rate was determined using the 95% confidence intervals estimated by the model.

The weekly results of the observed *versus* expected mortality rates are displayed graphically using z-scores [(number of observed deaths—expected mortality)/standard deviation of the residuals]. Z-scores ranging between -2 and +2 are considered ‘normal’, while a z-score >4 is considered a substantial increase.^30^^,^^31^

All analyses were performed in R Statistical Software, version 4.0.5 (The R Foundation for Statistical Computing, Vienna, Austria).

## Results

Quality of vital registrations between countries varied, with 17 countries (85.0%) having very high or high, one country (Peru) having medium, and two countries (Georgia and Ukraine) having low quality civil registration and vital statistics systems^32^ (Supplementary Table S1). Therefore, presented results need to be interpreted with analogous caution.

**Figure 1:**
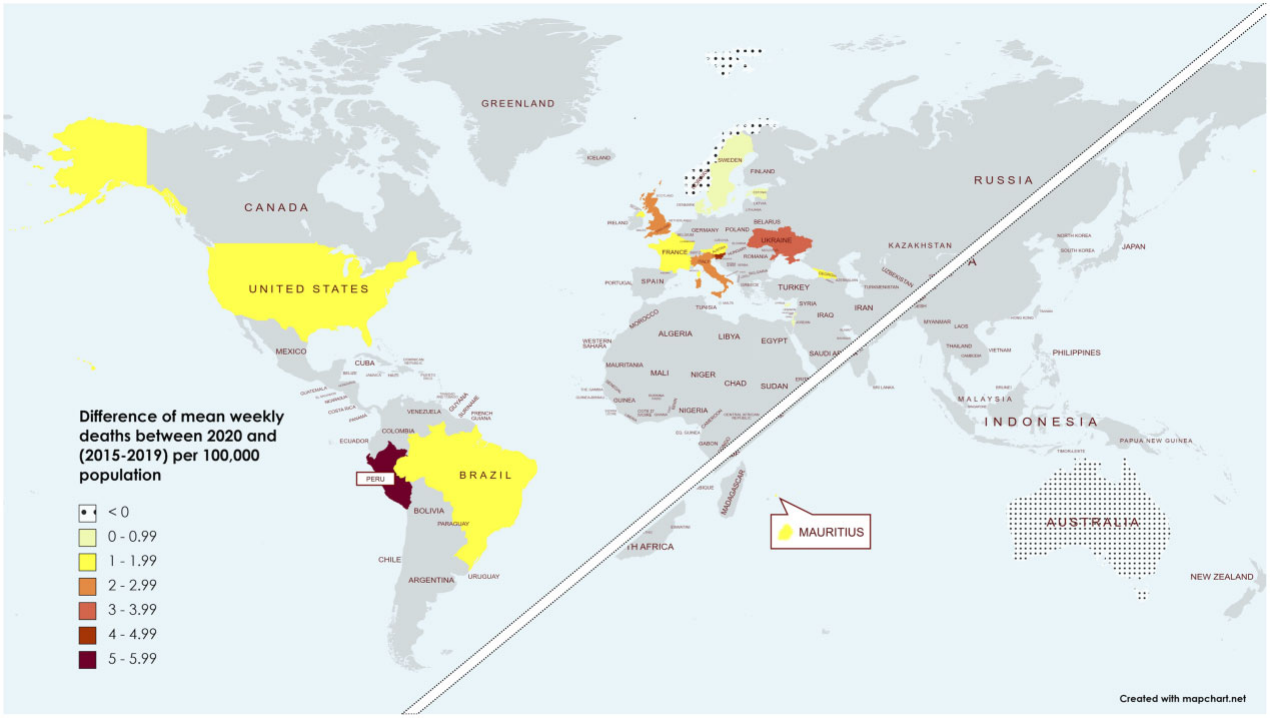
Difference in weekly mean of observed mortality rates between 2020 and 2015–19

The average weekly mortality rate per 100 000 varied widely between years and between countries (Supplementary Table S3, available as Supplementary data at *IJE* online). Figure 1 displays the difference in weekly mean of all-cause deaths between years 2015–2019 and year 2020 for each country, per 100 000 population (raw data in Supplementary Table S3). All participating countries, except Australia and Norway, experienced a higher weekly mean of all-cause mortality rate in 2020 than during the previous five years.

### Weekly comparisons—total population and by sex

**Figure 2:**
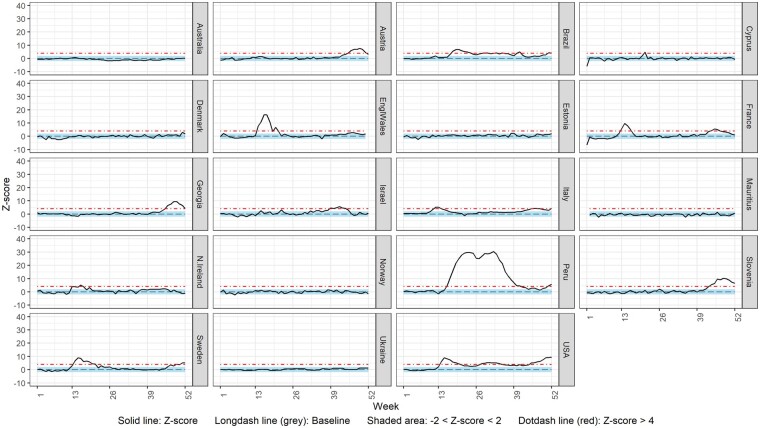
Weekly z-score of age-standardized all-cause mortality rate for total population

Figure 2 shows the weekly ASMR z-score over time from Week 1 2020 to Week 52 2020 for the total population for each country with the exception of Scotland, for which ASMR could not be estimated. Of the 19 included countries, Austria (Weeks 45–51), Brazil (Weeks 18–24, 29–30, 32–33, 35, 41 and 51–52), Cyprus (Week 21), England and Wales (Weeks 14–18, 20), France (Weeks 13–16 and 44–47), Georgia (Weeks 46–52), Israel (Weeks 39–44), Italy (Weeks 12–14, 45–47 and 52), Northern Ireland (Weeks 13 and 16), Peru (Weeks 16–41 and 51–52), Slovenia (Weeks 43–52), Sweden (Weeks 14–21, 49 and 51–52) and the USA (Weeks 14–20, 28–34, and 45–52) displayed substantial (>4 z-scores) excess mortality in 2020. In contrast, Australia, Denmark, Estonia, Mauritius, Norway and Ukraine did not display substantial excess mortality for any week during 2020. Similar results were obtained when analysing CMRs; CMR analysis for Scotland highlighted substantial excess mortality in Weeks 14–20 (Supplementary Figure S1, available as Supplementary data at *IJE* online).

**Figure 3:**
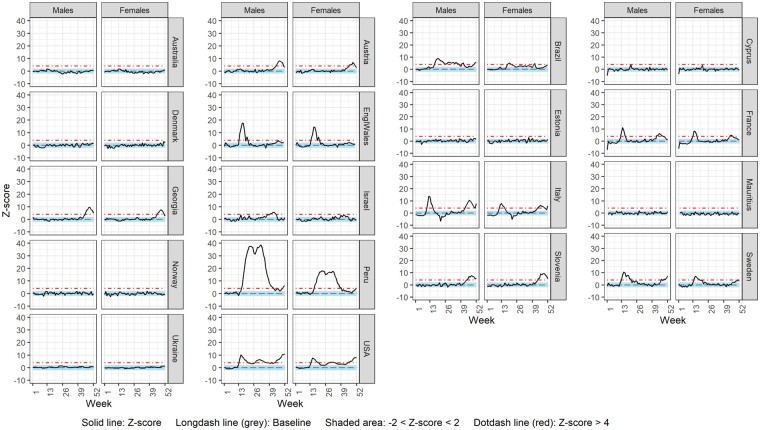
Weekly z-score of age-standardized all-cause mortality rate by sex

Sex-specific weekly ASMR z-scores over 2020 are shown for each country in Figure 3, except for Northern Ireland and Scotland for which sex-specific all-cause mortality and ASMR were not available, respectively (Supplementary Table S1). For most countries displaying substantial excess mortality, this was higher in males compared with females (Austria, Brazil, England and Wales, France, Georgia, Israel, Italy, Peru, Sweden and the USA) for the majority of weeks. Only Slovenia experienced higher excess mortality in females compared with males. In Cyprus, there were no notable differences in excess mortality between sexes. Similar trends were observed using CMRs, with the exception of the USA for which CMRs were higher in females than in males; CMR analysis for Scotland showed higher excess mortality in males than in females (Supplementary Figure S2, available as Supplementary data at *IJE* online).

### Weekly comparisons—by age group

**Figure 4:**
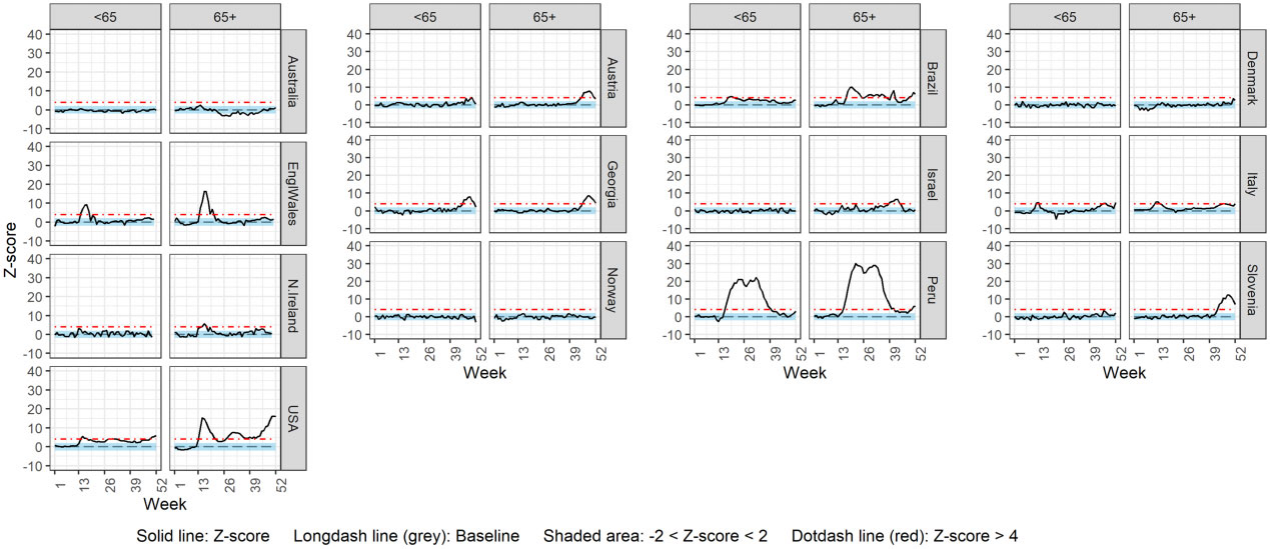
Weekly z-score of all-cause mortality rate for age groups <65 and 65+

**Figure 5:**
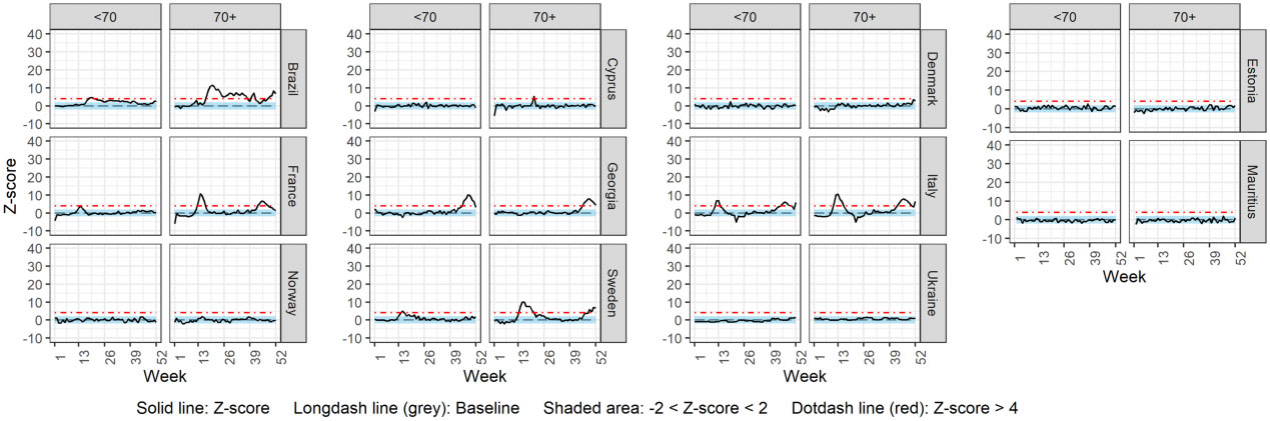
Weekly z-score of all-cause mortality rate for age groups <70 and 70+

Figures 4 and 5 display weekly mortality rate z-score over time for ages <65 vs 65+ and ages <70 vs 70+ years, respectively. Countries were included in each figure, or in both, according to the age breakdown of the data provided. Scotland was not included due to lack of age-specific all-cause mortality data (Supplementary Table S1). In Austria, Cyprus, Israel and Slovenia, substantial excess mortality was only observed in the oldest age group, whereas for Brazil, England and Wales, France, Georgia, Italy, Northern Ireland, Sweden, Peru and the USA, substantial excess mortality was observed in both age groups but was more pronounced in the oldest one (65+ and/or 70+ years). For Estonia, even though excess mortality was not substantial for either age group, it was more pronounced in the <70 age group than in the 70+ age group.

Supplementary Figure S3 (available as Supplementary data at *IJE* online), displays the observed z-scores against those expected by more detailed age breakdowns for the countries observing excess mortality in the younger age groups. If substantial excess mortality was observed in the <65 years age group, this was further divided into <45 and 45–64 years (Supplementary Figure S3a), and the <70 years age group was further broken down to <50 and 50–69 years (Supplementary Figure S3b). For the USA, the available age groups were <15 and 15–64 years (Supplementary Figure S3c, available as Supplementary data at *IJE* online). Most countries observed substantial excess mortality only in the older of the two age groups. In contrast, Peru demonstrated substantial excess mortality also in the <45 years old age group.

### Excess mortality in 2020

**Figure 6:**
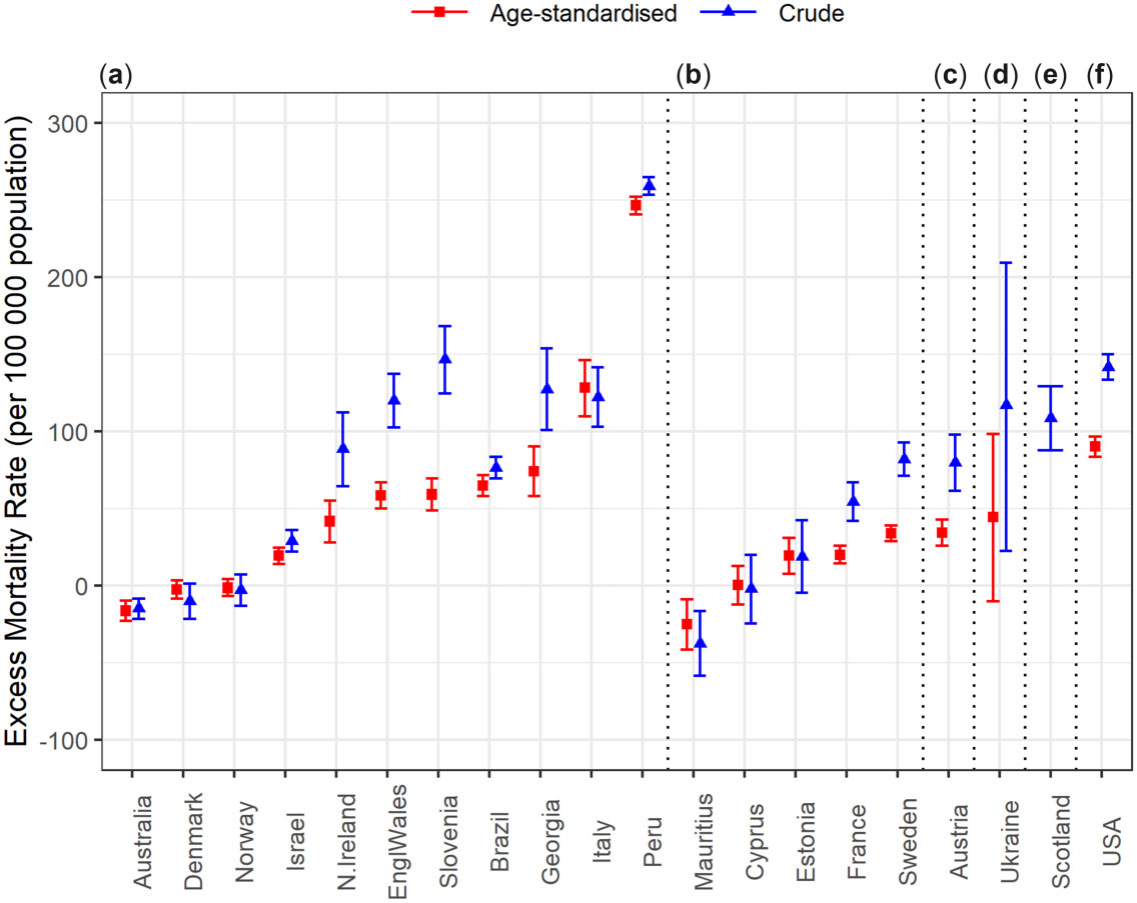
Cumulative excess crude and age-standardized mortality rate, for 2020. Plot letters correspond to the age groups in which countries have provided data and therefore the age groups used for age standardization: (a) age groups <15, 15–44, 45–64, 65+; (b) age groups <19, 20–49, 50–69, 70+; (c) age groups <19, 20–49, 50–64, 65+; (d) age groups <15, 15–64, 65+; (e) age groups <19, 20–54, 55–69, 70+ (Supplementary Table S3, available as Supplementary data at *IJE* online)

Figure 6 and Supplementary Table S4 (available as Supplementary data at *IJE* online) display the cumulative expected and observed all-cause mortality rates for the whole year (2020), using CMRs and ASMRs. The following countries demonstrated statistically significant excess cumulative ASMRs during 2020: Austria, Brazil, France, England and Wales, Estonia, Georgia, Italy, Israel, Northern Ireland, Peru, Slovenia, Sweden and the USA. Scotland also demonstrated excess CMR in 2020. On the other hand, Australia and Mauritius demonstrated statistically significant decreases in yearly all-cause mortality rate. Cyprus, Denmark, Norway and Ukraine, observed no statistically significant differences in all-cause mortality. Similar results were obtained with CMR, except for Estonia, where the increase in CMR is not significant, and for Ukraine, where the increase in CMR is significant in contrast to the increase in ASMR.

Supplementary Tables S5 and S6 (available as Supplementary data at *IJE* online) report the yearly cumulative mortality rate differences (observed-expected) by sex using CMRs and ASMRs, respectively. Sex-specific differences in all-cause ASMR for the whole year were only observed for Norway and Ukraine (significant increase only in males), and for Mauritius (statistically significant decrease only in females). Supplementary Table S7 (available as Supplementary data at *IJE* online) demonstrates the cumulative CMR differences (observed-expected) by age group. Age-specific differences in all-cause mortality for the whole year were observed in France, Israel and Slovenia (significant increase only in the older age group: 65+ or 70+), in Estonia (significant increase only in the younger age group: <70) and in Ukraine (significant decrease in the younger age group and significant increase in the older age group: 70+).

## Discussion

This study evaluated total, sex-specific and age-specific excess all-cause mortality in 20 countries during the year 2020.

The majority of investigated countries (Austria, Brazil, Cyprus, England and Wales, France, Georgia, Israel, Italy, Northern Ireland, Peru, Scotland, Slovenia, Sweden and the USA) displayed significant excess mortality during at least 1 week of 2020. The duration of the significant excess mortality varied widely, between 1 and 2 weeks in Cyprus and Northern Ireland, 6 to 8 weeks in Austria, England and Wales, Georgia, Israel, Italy and Scotland, nine to 11 weeks in France, Slovenia, and Sweden, 15 weeks in Brazil, 28 weeks in Peru and 37 weeks in the USA. Conversely, Australia, Denmark, Estonia, Mauritius, Norway and Ukraine did not observe significant excess all-cause mortality, compared with what was expected, for any week in 2020. These results are largely in agreement with results published elsewhere, despite the use of varying methodologies and data sources,^19^^,^^33–37^ thus reinforcing our findings.

More specifically, Schöley *et al.* (2021), comparing varying methodologies using data from the Short Term Mortality Fluctuations (STMF) database, also identified Slovenia as having a high excess of all-cause deaths, Denmark and Norway with very little and Austria and Sweden in the middle ranks of excess all-cause mortality.^37^ Using the same data, Islam *et al.* (2021) also identified the USA, Italy and England and Wales as being the most affected countries in terms of excess deaths, and Slovenia, the USA, countries of the UK and Italy as most affected in terms of ASMRs. Karlinsky and Kobak (2021) used data from the World Mortality Dataset until the end of 2020 or the first half of 2021, and also evidenced Peru, Brazil, the USA, Italy and countries of the UK to be most affected and Australia to be the least affected in terms of excess absolute death numbers.^38^ Nepomuceno *et al*. (2021) identified Italy, the USA, Slovenia and countries of the UK as the most heavily affected in terms of CMRs and ASMRs, using an array of different methodologies.^39^ Last, in their investigation of Latin American countries, Lima *et al.* (2021) also identified Peru and Brazil as having experienced high excess all-cause mortality.^40^

As expected, the countries with prolonged substantial excess mortality in the weekly comparison also demonstrated statistically significant cumulative excess ASMR when comparing the sum of observed weekly mortality rates with the sum of expected weekly mortality rates for 2020, with the exception of Estonia. Despite having zero weeks with substantial (z-score >4) excess mortality, the statistically significant, but small in magnitude, excess yearly mortality observed by Estonia could be explained by the relatively stable mortality pattern of the country, which made even small increases in mortality show up as statistically significant.

The observed pattern of weekly excess mortality in the investigated countries indicates that some countries experienced substantial excess mortality during the first half of the year but not later (Cyprus, England and Wales, Northern Ireland and Scotland), whereas others did so during the last trimester of 2020 but not earlier (Austria, Georgia, Israel and Slovenia). For some countries, two distinct peaks in excess mortality were observed suggesting two pandemic mortality waves (France, Italy and Sweden), whereas for the most affected countries substantial excess mortality was prolonged throughout the year (Brazil, Peru and the USA). Similar peaks in excess mortality for the participating countries were observed elsewhere.^19^^,^^33–36^ Variation in the timing, strictness and duration of governmental control measures could explain the excess mortality patterns in the participating countries,^7^ along with other indicators shown to influence excess mortality such as health privatization, health expenditure, numbers of doctors and hospital beds, share of population covered by health insurance and test-and-trace capacity.^41^

In sex-specific weekly analyses, excess ASMR was more pronounced in males than in females, with the exception of Slovenia (higher in females) and of Cyprus (similar in both sexes). In the yearly cumulative comparison, increases or decreases in all-cause mortality were similar between sexes except for Norway (statistically significant increase only in males), for Mauritius (statistically significant decrease only in females) and for Ukraine (statistically significant increase in males and decrease in females). The male predominance in excess mortality observed in most countries, even following standardization, was previously shown^19^^,^^42^^,^^43^ and is expected, since male sex was identified as a risk factor for death by a global COVID-19 meta-analysis.^44^ In fact, all-cause male mortality has been historically higher compared with female all-cause mortality.^45^ Biological factors, such as a stronger immune response, and behavioural risk factors, such as smoking and other lifestyle habits, are some of the reasons why men are at a greater risk for death as a consequence of COVID-19 or of other common causes of death.^46^ In Slovenia the excess mortality in females is probably explained by the observed higher case fatality ratio for COVID-19 among females than males.^47^ Taken together, these results support the need for sex-specific investigations in excess mortality to better assess and understand the determinants of the true toll of the COVID-19 pandemic.

Interestingly, in total and sex-specific analyses, similar results were obtained when analysing CMR instead of ASMRs, except for Estonia, where the increase in cumulative 2020 CMR is not significant, and of Ukraine, where the increase in CMR is significant in contrast to the increase in ASMR. Another exception is sex-specific excess mortality in the USA, for which CMR was higher in females than in males, whereas ASMR was higher in males than in females. These results suggest that the age pattern of mortality was different across time within countries and also between the sexes, supporting the use of ASMRs where possible.^30^

With respect to weekly age-specific analyses, for most countries excess mortality was only evident (Austria, Cyprus, Israel and Slovenia) or was higher (Brazil, England and Wales, France, Georgia, Italy, Northern Ireland, Sweden, Peru and the USA) in the oldest age group investigated (65+ or 70+). This finding is expected since, in the absence of vaccination, age is one of the strongest predictors of all-cause and COVID-19 mortality^48^ and globally, the age dependency of COVID-19 (the factor by which the risk of COVID-19 mortality increases if age is increased by 10 years) is strongly associated with all-cause mortality.^49^ Interestingly, for Peru, excess cumulative mortality for the whole of 2020 was substantially high also in the age group <45. This result is supported by other studies showing that countries in the Americas have suffered the most potential years of life lost due to COVID-19.^42^ Demographic, socioeconomic, racial and pre-pandemic health status factors, such as comorbidity prevalence, can explain the increased risk of death from both COVID-19 and other causes in younger age groups in this country.^16^^,^^50–52^

The comparison of the sum of observed deaths with the sum of expected deaths for the whole year (2020) yielded mostly similar results, with some surprising findings. In Estonia the significant increase in yearly deaths was only observed in the younger age group (<70). This result can be explained by an increased prevalence of comorbidities in the Estonian population aged under 70 years^53^ and by strict control measures taken, which directly or indirectly protected the most vulnerable age groups during 2020.^54^

### Strengths and limitations

To our knowledge, this is one of few studies investigating total, sex-specific and age-specific excess mortality for a diverse group of countries, relying on national data sources for mortality estimates. It complements other studies that quantify the impacts of the COVID-19 pandemic through life expectancy losses.^18^^,^^40^ In addition, it is the first peer-reviewed publication of excess mortality results for Mauritius and Georgia. Of the 20 countries included in the analysis, 17 (85.0%) were evaluated as having very high- or high-quality civil registration and vital statistics systems; only two (Georgia and Ukraine) and one (Peru) countries were evaluated as having low- and medium-quality systems, respectively).^32^ Focusing on death registrations, all countries included in this investigation had a coverage of 90% and above, except for Peru (50–74%).^55^ In addition, the model used for the estimation of excess mortality is one that has been shown to produce estimates with the least bias compared with other methods.^37^^,^^39^ Furthermore, this investigation allowed for any delays in data reporting, ensuring a more accurate representation of the mortality experience of countries during 2020. Of note, as the analysis was carried out using data from 2020, results were not influenced by the effects of vaccination (scarcely available in any country before the end of 2020; highest population percentages fully vaccinated on 31 December 2020 were 0.6% and 0.01% for the UK and the USA, respectively^48^) nor by newer virus variants. Therefore, the results serve as a useful comparator against which to investigate the effects of vaccinations and impact of new variants which defined the pandemic trajectory in subsequent years. However, our study also has some limitations such as the lack of data that would allow estimation of the direct and indirect contributions of COVID-19 to excess all-cause mortality. In addition, participating countries did not use consistent age groups to calculate age-specific all-cause mortality. Age-standardized results are thus not fully comparable between countries. Therefore, the magnitude of excess mortality for 2020 should not be used as a measure of comparison of impact between countries; rather as an indicator of the COVID-19 impact on all-cause mortality in each country. Last, even though the high quality of registration systems in the majority of the countries included in our investigation reinforces the validity of our results, it is important to acknowledge that for countries where the health system was overwhelmed with hospitalizations and mortality during 2020, quality could be compromised to a degree not amenable to allowing sufficient time to capture any delays in data reporting.

## Conclusion

Our findings on excess mortality during 2020 show the asymmetrical impacts of the pandemic, highlighting countries where the impact was more extensive or more limited. Overall, males carried a heavier burden, with the exception of Slovenia where females displayed a higher excess mortality than males. In most countries, excess mortality was substantial and a public health concern in the oldest age groups, with some notable exceptions, namely Peru where excess mortality was high also in younger age groups, and Estonia where excess mortality for the whole year was higher in the 50–69 group compared with the 70+ group.

These results, which show that excess mortality during the first year of the pandemic was context-specific, prompt further investigation into the determinants of excess mortality in countries and in specific sex and age groups, which will further suggest steps to strengthen health resilience for those most affected. Furthermore as the pandemic continues, tracking excess mortality is of paramount importance in order to accurately estimate the true toll of COVID-19, at the same time investigating the effects that different variants, vaccination strategies and further public health interventions had in the studied countries.

## Ethics approval

Ethical approval to conduct the study was obtained from the Cyprus National Bioethics Committee (16/6/2020, ΕΕΒΚ/ΕΠ/2020/01.127).

## Supplementary Material

dyac170_Supplementary_Data

## Data Availability

The data underlying this study, beyond what is available in the article and in its online Supplementary material, can be shared to facilitate methodologically sound proposals after signing a data access agreement. Proposals and data requests should be directed to [demetriou.chri@unic.ac.cy].
