# Correction: Impact of the COVID-19 pandemic on total, sex- and age-specific all-cause mortality in 20 countries worldwide during 2020: results from the C-MOR project

**DOI:** 10.1093/ije/dyae001

**Published:** 2024-01-12

This is a correction to: Christiana A Demetriou, Souzana Achilleos, Annalisa Quattrocchi, John Gabel, Elena Critselis, Constantina Constantinou, Nicoletta Nicolaou, Giuseppe Ambrosio, Catherine M Bennett, Nolwenn Le Meur, Julia A Critchley, Laust Hvas Mortensen, Jose Manuel Rodriguez-Llanes, Mario Chong, Gleb Denissov, Petra Klepac, Lucy P Goldsmith, Antonio José Leal Costa, Terje P Hagen, Marie Chan Sun, Qian Huang, Nataliia Pidmurniak, Inbar Zucker, Joseph Cuthbertson, Bo Burström, Manuel Barron, Ivan Eržen, Fabrizio Stracci, Wilson Calmon, Cyndy Martial, Olesia Verstiuk, Zalman Kaufman, Wenjing Tao, Maia Kereselidze, Nino Chikhladze, Claudia Zimmermann, Eva Schernhammer, Antonis Polemitis, Andreas Charalambous, on behalf of the C-MOR Consortium, Impact of the COVID-19 pandemic on total, sex- and age-specific all-cause mortality in 20 countries worldwide during 2020: results from the C-MOR project, *International Journal of Epidemiology*, Volume 52, Issue 3, June 2023, Pages 664–676, https://doi.org/10.1093/ije/dyac170

In the originally published version of this article, two names were missed from the authorship list. Towards the end of the authorship list, the additional names and affiliation should now read:

“[…], Nino Chikhladze^30^, Claudia Zimmermann^31^, Eva Schernhammer^31^, Antonis Polemitis^32^, and Andreas Charalambous^2^[…]” and “[…]^30^Faculty of Medicine, Ivane Javakhishvili Tbilisi State University, Tbilisi, Georgia,^31^Department of Epidemiology, Center for Public Health, Medical University of Vienna, Vienna, Austria, and ^32^ University of Nicosia, Nicosia, Cyprus”.

instead of:

“[…], Nino Chikhladze^30^, Antonis Polemitis^31^, and Andreas Charalambous^2^[…]” and “[…]^30^Faculty of Medicine, Ivane Javakhishvili Tbilisi State University, Tbilisi, Georgia and ^31^University of Nicosia, Nicosia, Cyprus”.

The missing author names and affiliation have been emended within the article online only.

