# Supplementary material for: Impact of the COVID-19 pandemic on total, sex- and age-specific all-cause mortality in 20 countries worldwide during 2020: results from the C-MOR project
Source: Int J Epidemiol. 2022 Aug 27;52(3):664–76. doi: 10.1093/ije/dyac170 (PMC9452146; doi:10.1093/ije/dyac170)

**Supplementary Material**

**Excess total, sex- and age-specific all-cause mortality in 20 countries during 2020: a time series analysis using data from national primary sources**

**Supplementary Methods: Equations for the Statistical Analysis**

$${Crude mortality rate (CMR)}_{y, w}= \frac{D_{y,w}}{P_{y} / N_{w}} \times100,000 (1)$$

where D*_y,w_* = number of deaths in all age-groups in one week, P=mid-year population, and N*_w_*= number of weeks in the year.

$${Age specific mortality rate (ASpDR)}_{y, w, i}= \frac{D_{y,w,i}}{P_{y,i} / N_{w}} \times100,000 (2)$$

where D*_y,w,i_* = number of deaths in the age-group $i$ in one week, P=mid-year population in age group i , and N*_w_*= number of weeks in the year.

$$Age-standardised mortality rate (ASMR)=\sum_{i=1}^{n} \left( p_{i}^{s} {ASpDR}_{y,w,i} \right)(3)$$

where, index $i$ denotes the aggregate age groups (see Supplementary Table S2) and the standard population weights 𝑝^𝑖^_𝑠_ correspond to the respective broad age intervals in the WHO World Standard Population 2000-2025

**Table S1 - Summary of national data sources, period of available mortality data, time unit, availability of sex and age-specific data, and data quality of civil registration and vital statistics systems per country**

| **Country** | **Partners** | **All cause deaths (2015-2020)** | | | | |  |  |  |  |
| --- | --- | --- | --- | --- | --- | --- | --- | --- | --- | --- |
|  |  | **Access date** | **Source** | **Public data (Y/N)** | **Link (if available)** | **Notes** | **Time unit** | **Weekly sex specific data available** | **Weekly age specific data available** | **Data quality of civil registration and vital statistics systems*** |
| **Australia** | Deakin University; Monash University | Latest - October 2021 | Australian Bureau of Statistics Provisional Mortality Statistics | Yes | [Link](https://www.abs.gov.au/statistics/health/causes-death/provisional-mortality-statistics) |  | ISO | YES | YES | Very High |
| **Austria** | Center of Public Health, Medical University of Vienna | July 21st, 2021 | Cause of death statistics by Statistics Austria | No |  |  | ISO | YES | YES | Very High |
| **Brazil** | Universidade Federal do Rio de Janeiro; Fluminense Federal University | July 9th 2021 | National Mortality Information System (Sistema de Informações sobre Mortalidade - SIM) | Yes | [Link](http://svs.aids.gov.br/dantps/centrais-de-conteudos/dados-abertos/sim/) |  | Epi | YES | YES | Medium - High |
| **Cyprus** | University of Nicosia | September 2021 | Cyprus death registry, Health Monitoring Unit, Ministry of Health | Yes |  |  | ISO | YES | YES | High |
| **Denmark** | Statistics Denmark | July 7th 2021 | Statistics Denmark | Yes |  |  | Epi | YES | YES | High |
| **England and Wales** | St George's, University of London | April 13th 2021 | Office for National Statistics | Yes | [Link](https://www.ons.gov.uk/peoplepopulationandcommunity/birthsdeathsandmarriages/deaths/datasets/weeklyprovisionalfiguresondeathsregisteredinenglandandwales) |  | National | YES | YES | Very High |
| **Estonia** | National institute for Health Development | June 30th 2021 | Estonian Causes of Death Registry | Yes | [Link](https://statistika.tai.ee/pxweb/en/Andmebaas/Andmebaas__01Rahvastik__04Surmad/?tablelist=true) |  | ISO | YES | YES | Very High |
| **France** | EHESP, CNRS, ARENES - UMR 6051 | June 2021 | Institut National de la Statistique et des Etudes Economiques (INSEE) | Yes | [Link](https://www.insee.fr/fr/statistiques/4487988) |  | ISO | YES | YES | High-  Very High |
| **Georgia** | Tbilisi state university; National Center for Disease Control and Public Health | 2021 | Vital Registration system | Yes | [Link](https://www.geostat.ge/) |  | ISO | YES | YES | Low-Medium |
| **Israel** | Ministry of Health | Feb 1st 2021 | Ministry of Health Israel | No |  |  | Epi | YES | YES | High-  Very High |
| **Italy** | University of Perugia | May 18th 2021 | ISTAT (Italian Institute of Statistics) | Yes |  | Acknowledge ISS support. | Epi | YES | YES | Medium – High |
| **Mauritius** | University of Mauritius; Statistics Mauritius |  | Statistics Mauritius | No |  | Data can be provided on request. | National | YES | YES | High |
| **Northern Ireland** | St George's, University of London | May 18th 2021 | Northern Ireland Statistics and Research Agency | Yes | [Link](https://www.nisra.gov.uk/publications/historical-weekly-deaths-data) |  | National | NO | YES | Very High |
| **Norway** | University of Oslo | September 2021 | Statistics Norway | Yes | [Link](https://www.ssb.no/en/statbank/list/dode) |  | ISO | YES | YES | Very High |
| **Peru** | Universidad del Pacífico | 2020-2021 | Ministerio de Salud-Centro Nacional de Epidemiología, Prevención y Control de Enfermedades | Yes |  |  | ISO | YES | YES | Medium |
| **Scotland** | St George's, University of London | April 13th 2020 | National Records of Scotland | Yes | [Link](https://www.nrscotland.gov.uk/statistics-and-data/statistics/statistics-by-theme/vital-events/general-publications/weekly-and-monthly-data-on-births-and-deaths/weekly-data-on-births-and-deaths) | We used "weekly-march-21-tab-2" for the historical all-cause-mortality data. At the time of writing (23/12/21) "weekly-november-21-tab-2" is available on this link. Death by cause by week for respiratory, cancer, circulatory (I100-I99) and Influenza and pneumonia (J09-J18) was supplied on request by the National Records of Scotland on 15 April 2021. | ISO | YES | NO | Very High |
| **Slovenia** | National Institute of Public Health | July 29th 2021 | Central Registry of Patient Data | No |  | The data on all-cause mortality in Slovenia are updated and sent to EuroMOMO network weekly. They are published on the website https://www.euromomo.eu/graphs-and-maps. As they are updated every week, there may be slight changes in numbers. | ISO | YES | YES | High-  Very High |
| **Sweden** | Karolinska Institutet | August 13th 2021 | Statistics Sweden |  | [Link](https://www.scb.se/hitta-statistik/sverige-i-siffror/manniskorna-i-sverige/doda-i-sverige/) | All-cause mortality statistics are publically available (including stratification by age and sex categories) but not as detailed as required by C-MOR. | ISO | YES | YES | High-  Very High |
| **United States** | University of South Carolina | July 23rd 2021 | Human Mortality Database/Max Planck, National Center for Health Statistics (NCHS) | Yes | [Link](http://www.ukrstat.gov.ua/) | Data before epi week 5 in 2020 are from the Human Mortality Database/Max Planck, and data after epi week 5 in 2020 (included) are from NCHS. | Epi | YES | YES | High-  Very High |
| **Ukraine** | Bogomolets National Medical University | June 19th-29th 2021 | State Statistics Service of Ukraine | Yes | [Link](https://data.cdc.gov/NCHS/Provisional-COVID-19-Deaths-by-Week-Sex-and-Age/vsak-wrfu) | Publicly available data was just for the months. | ISO | YES | YES | Low - Medium |

Abbreviations: ISO: International Organization for Standardization; Epi: epidemiological

* Source: Mikkelsen L, Phillips DE, AbouZahr C, et al. A global assessment of civil registration and vital statistics systems: monitoring data quality and progress. The Lancet. Elsevier; 2015 Oct 3;386(10001):1395–1406. Reference years: 2005-2012

**Table S2 – Description of aggregate age groups created for the age-standardization according to age-specific all-cause mortality data availability**

| Country | Age groups (a)  (<15, 15-44, 45-64, 65+) | Age groups (b)  (<19, 20-49, 50-69, 70+) | Age groups (c)  (<19, 20-49, 50-64, 65+) | Age groups (d)  (<15, 15--64, 65+) | Age groups (e)  (<19, 20-54, 55-69, 70+) |
| --- | --- | --- | --- | --- | --- |
| Australia | **x** |  |  |  |  |
| Austria |  |  | **x** |  |  |
| Brazil | **x** | **x** |  |  |  |
| Cyprus |  | **x** |  |  |  |
| Denmark | **x** | **x** |  |  |  |
| England and Wales | **x** |  |  |  |  |
| Estonia |  | **x** |  |  |  |
| France |  | **x** |  |  |  |
| Georgia | **x** | **x** |  |  |  |
| Israel | **x** |  |  |  |  |
| Italy | **x** | **x** |  |  |  |
| Mauritius |  | **x** |  |  |  |
| Northern Ireland |  |  |  |  |  |
| Norway | **x** | **x** |  |  |  |
| Peru | **x** |  |  |  |  |
| Scotland |  |  |  |  |  |
| Slovenia | **x** |  |  |  |  |
| Sweden |  | **x** |  |  |  |
| United States |  |  |  | **x** |  |
| Ukraine |  |  |  |  | **x** |

**Table S3 -** **Comparison of weekly observed mortality rates between 2015-2019 vs. 2020**

|  | **mean (SD) of weekly mortality rate (per 100,000 population)** | |
| --- | --- | --- |
| **Country** | **2015-2019** | **2020** |
| **Australia** | 11·0 (0·9) | 10·5 (0·5) |
| **Austria** | 18·1 (2·0) | 19·5 (3·5) |
| **Brazil** | 12·1 (0·6) | 13·7 (1·6) |
| **Cyprus** | 13·1 (2·3) | 13·4 (2·5) |
| **Denmark** | 17·8 (1·5) | 17·9 (1·3) |
| **England & Wales** | 17·4 (2·3) | 19·4 (5·1) |
| **Estonia** | 22·3 (2·4) | 22·6 (1·9) |
| **France** | 17·5 (1·8) | 18·9 (3·1) |
| **Georgia** | 24·7 (3·0) | 26·1 (6·2) |
| **Israel** | 10·0 (1·2) | 10·3 (1·0) |
| **Italy** | 20·6 (2·6) | 23·1 (5·7) |
| **Mauritius** | 15·5 (2·3) | 16·5 (1·8) |
| **Northern Ireland** | 16·1 (2·5) | 17·7 (2·8) |
| **Norway** | 14·8 (1·4) | 14·4 (1·1) |
| **Peru** | 6·5 (0·6) | 12·3 (5·3) |
| **Scotland** | 20·4 (2·5) | 22·4 (4·4) |
| **Slovenia** | 18·6 (2·5) | 22·5 (6·4) |
| **Sweden** | 17·0 (1·7) | 17·7 (2·8) |
| **Ukraine** | 16·5 (1·0) | 19·7 (2·3) |
| **USA** | 25·2 (7·0) | 27·1 (5·3) |

*SD, standard deviation.*

**Table S4 - Cumulative observed and expected mortality rates per 100,000 population for 2020; total population**

|  | **Crude mortality rate (CMR)** | | | | | | | **Age standardized mortality rate (ASMR)** | | | | | | | |
| --- | --- | --- | --- | --- | --- | --- | --- | --- | --- | --- | --- | --- | --- | --- | --- |
| **Country** | **Observed mortality rate / 100,000 population*** | **Expected mortality rate / 100,000 population *** | **Lower limit of 95% CI of Expected mortality rate** | **Upper limit of 95% CI of mortality rate** | **Difference (Observed-Expected mortality rate)** | **Difference using the Upper limit of 95% CI of Expected mortality rate** | **Difference using the Lower limit of 95% CI of Expected mortality rate** |  | **Observed mortality rate / 100,000 population*** | **Expected mortality rate / 100,000 population *** | **Lower limit of 95% CI of Expected mortality rate** | **Upper limit of 95% CI of mortality rate** | **Difference (Observed-Expected mortality rate)** | **Difference using the Upper limit of 95% CI of Expected mortality rate** | **Difference using the Lower limit of 95% CI of Expected mortality rate** |
| **Australia** | 548 | 563 | 556 | 569 | -15↓ | -22 | -8 |  | 305 | 321 | 315 | 328 | -16↓ | -23 | -10 |
| **Austria** | 1019 | 939 | 921 | 958 | 80↑ | 61 | 98 |  | 485 | 451 | 442 | 459 | 34↑ | 26 | 43 |
| **Brazil** | 716 | 640 | 633 | 647 | 76↑ | 69 | 83 |  | 634 | 570 | 563 | 576 | 65↑ | 58 | 71 |
| **Cyprus** | 698 | 700 | 678 | 722 | -2 | -25 | 20 |  | 377 | 377 | 365 | 390 | 0.2 | -12 | 13 |
| **Denmark** | 930 | 940 | 928 | 952 | -10 | -22 | 1 |  | 437 | 439 | 434 | 445 | -3 | -9 | 3 |
| **England & Wales** | 1012 | 892 | 875 | 909 | 120↑ | 102 | 137 |  | 512 | 454 | 445 | 462 | 58↑ | 50 | 67 |
| **Estonia** | 1171 | 1152 | 1129 | 1176 | 19 | -5 | 42 |  | 564 | 545 | 533 | 556 | 19↑ | 8 | 31 |
| **France** | 980 | 926 | 913 | 938 | 54↑ | 42 | 67 |  | 433 | 413 | 408 | 419 | 20↑ | 14 | 25 |
| **Georgia** | 1351 | 1223 | 1197 | 1250 | 127↑ | 101 | 154 |  | 834 | 760 | 744 | 777 | 74↑ | 58 | 90 |
| **Israel** | 532 | 503 | 496 | 510 | 29↑ | 22 | 36 |  | 388 | 369 | 363 | 374 | 19↑ | 14 | 25 |
| **Italy** | 1195 | 1073 | 1054 | 1093 | 122↑ | 103 | 141 |  | 472 | 343 | 325 | 362 | 128↑ | 110 | 146 |
| **Mauritius** | 869 | 906 | 885 | 927 | -38↓ | -59 | -17 |  | 647 | 672 | 656 | 688 | -25↓ | -42 | -9 |
| **Northern Ireland** | 921 | 832 | 809 | 856 | 88↑ | 64 | 112 |  | 512 | 471 | 457 | 484 | 42↑ | 28 | 55 |
| **Norway** | 748 | 751 | 741 | 761 | -3 | -13 | 7 |  | 391 | 393 | 387 | 398 | -2 | -7 | 4 |
| **Peru** | 639 | 380 | 374 | 386 | 259↑ | 253 | 265 |  | 614 | 368 | 362 | 373 | 246↑ | 241 | 252 |
| **Scotland** | 1165 | 1056 | 1035 | 1077 | 108↑ | 87 | 129 |  | - | - | - | - | - | - | - |
| **Slovenia** | 1159 | 1013 | 991 | 1035 | 146↑ | 125 | 168 |  | 524 | 465 | 455 | 476 | 59↑ | 49 | 69 |
| **Sweden** | 913 | 832 | 821 | 842 | 82↑ | 71 | 93 |  | 383 | 349 | 344 | 354 | 34↑ | 29 | 39 |
| **Ukraine** | 1398 | 1281 | 1188 | 1375 | 117↑ | 22 | 209 |  | 782 | 738 | 684 | 793 | 44 | -10 | 98 |
| **USA** | 1018 | 876 | 868 | 884 | 142↑ | 133 | 150 |  | 643 | 553 | 547 | 560 | 90↑ | 83 | 96 |

*For all countries, the sum of observed and expected deaths is up to week 52, with the exception of England & Wales and Scotland (up to week 51), N. Ireland (up to week 50), and Mauritius for weeks 2-52

^↑^ Indicates statistically significant excess all-cause mortality using the sum of deaths for the whole of 2020

^↓^ Indicates a statistically significant reduction all-cause mortality using the sum of deaths for the whole of 2020

**Table S5 – Comparison of the cumulative observed and expected mortality rate for the whole year (2020); crude mortality rate (CMR) by sex**

|  | **Males** | | | | | | |  | **Females** | | | | | | |
| --- | --- | --- | --- | --- | --- | --- | --- | --- | --- | --- | --- | --- | --- | --- | --- |
| **Country** | **Observed mortality rate / 100,000 population*** | **Expected mortality rate / 100,000 population *** | **Lower limit of 95% CI of Expected mortality rate** | **Upper limit of 95% CI of mortality rate** | **Difference (Observed-Expected mortality rate)** | **Difference using the Lower limit of 95% CI of Expected mortality rate** | **Difference using the Upper limit of 95% CI of Expected mortality rate** |  | **Observed mortality rate / 100,000 population*** | **Expected mortality rate / 100,000 population *** | **Lower limit of 95% CI of Expected mortality rate** | **Upper limit of 95% CI of mortality rate** | **Difference (Observed-Expected mortality rate)** | **Difference using the Lower limit of 95% CI of Expected mortality rate** | **Difference using the Upper limit of 95% CI of Expected mortality rate** |
| **Australia** | 554 | 569 | 562 | 575 | -15↓ | -21 | -8 |  | 541 | 556 | 550 | 563 | -15↓ | -22 | -9 |
| **Austria** | 1024 | 933 | 915 | 952 | 90↑ | 72 | 108 |  | 1014 | 945 | 925 | 966 | 69↑ | 49 | 90 |
| **Brazil** | 817 | 718 | 711 | 726 | 99↑ | 92 | 106 |  | 617 | 563 | 557 | 570 | 54↑ | 47 | 60 |
| **Cyprus** | 751 | 756 | 727 | 786 | -5 | -35 | 24 |  | 646 | 646 | 617 | 675 | 0 | -29 | 29 |
| **Denmark** | 958 | 966 | 952 | 981 | -8 | -22 | 6 |  | 901 | 914 | 901 | 928 | -13 | -27 | 1 |
| **England & Wales** | 1035 | 908 | 892 | 924 | 127↑ | 111 | 143 |  | 989 | 876 | 858 | 895 | 112↑ | 93 | 131 |
| **Estonia** | 1169 | 1139 | 1110 | 1169 | 30↑ | 0 | 59 |  | 1173 | 1164 | 1136 | 1193 | 9 | -20 | 37 |
| **France** | 1015 | 952 | 941 | 964 | 62↑ | 50 | 74 |  | 948 | 901 | 887 | 915 | 47↑ | 33 | 61 |
| **Georgia** | 1469 | 1318 | 1290 | 1347 | 151↑ | 122 | 179 |  | 1243 | 1137 | 1107 | 1167 | 106↑ | 76 | 135 |
| **Israel** | 548 | 510 | 502 | 519 | 38↑ | 29 | 46 |  | 516 | 496 | 487 | 505 | 20↑ | 11 | 29 |
| **Italy** | 1195 | 1059 | 1042 | 1076 | 135↑ | 118 | 152 |  | 1196 | 1086 | 1065 | 1108 | 110↑ | 88 | 132 |
| **Mauritius** | 985 | 1000 | 971 | 1028 | -15 | -44 | 14 |  | 755 | 816 | 791 | 841 | -60↓ | -85 | -35 |
| **Norway** | 731 | 727 | 715 | 740 | 4 | -8 | 16 |  | 765 | 775 | 761 | 789 | -10 | -24 | 4 |
| **Peru** | 770 | 410 | 404 | 417 | 360↑ | 353 | 366 |  | 510 | 350 | 344 | 356 | 160↑ | 154 | 166 |
| **Scotland** | 1196 | 1073 | 1052 | 1094 | 123↑ | 101 | 144 |  | 1135 | 1045 | 1020 | 1071 | 90↑ | 65 | 115 |
| **Slovenia** | 1143 | 1006 | 980 | 1033 | 137↑ | 110 | 163 |  | 1176 | 1019 | 993 | 1046 | 156↑ | 130 | 183 |
| **Sweden** | 909 | 811 | 800 | 823 | 97↑ | 86 | 109 |  | 918 | 852 | 838 | 865 | 66↑ | 53 | 80 |
| **Ukraine** | 1651 | 1405 | 1269 | 1546 | 246↑ | 105 | 382 |  | 1179 | 1173 | 1058 | 1292 | 5 | -113 | 120 |
| **USA** | 1076 | 1006 | 997 | 1015 | 70↑ | 62 | 79 |  | 961 | 749 | 742 | 757 | 211↑ | 204 | 219 |

Northern Ireland is excluded due to the lack of sex-specific all-cause mortality data

*For all countries, the sum of observed and expected deaths is up to week 52, with the exception of England & Wales and Scotland (up to week 51), and Mauritius for weeks 2-52

^↑^ Indicates statistically significant excess all-cause mortality using the sum of deaths for the whole of 2020

^↓^ Indicates a statistically significant reduction all-cause mortality using the sum of deaths for the whole of 2020

**Table S6 – Comparison of the cumulative observed and expected mortality rate for the whole year (2020); age-standardized mortality rate (ASMR) by sex**

|  | **Males** | | | | | | |  | **Females** | | | | | | |
| --- | --- | --- | --- | --- | --- | --- | --- | --- | --- | --- | --- | --- | --- | --- | --- |
| **Country** | **Observed mortality rate / 100,000 population*** | **Expected mortality rate / 100,000 population *** | **Lower limit of 95% CI of Expected mortality rate** | **Upper limit of 95% CI of mortality rate** | **Difference (Observed-Expected mortality rate)** | **Difference using the Lower limit of 95% CI of Expected mortality rate** | **Difference using the Upper limit of 95% CI of Expected mortality rate** |  | **Observed mortality rate / 100,000 population*** | **Expected mortality rate / 100,000 population *** | **Lower limit of 95% CI of Expected mortality rate** | **Upper limit of 95% CI of mortality rate** | **Difference (Observed-Expected mortality rate)** | **Difference using the Lower limit of 95% CI of Expected mortality rate** | **Difference using the Upper limit of 95% CI of Expected mortality rate** |
| **Australia** | 327 | 333 | 328 | 338 | -6↓ | -11 | -1 |  | 264 | 269 | 265 | 274 | -6↓ | -10 | -1 |
| **Austria** | 547 | 503 | 493 | 512 | 44↑ | 34 | 54 |  | 430 | 404 | 395 | 412 | 26↑ | 17 | 35 |
| **Brazil** | 788 | 691 | 684 | 698 | 97↑ | 89 | 104 |  | 504 | 462 | 456 | 468 | 42↑ | 36 | 48 |
| **Cyprus** | 440 | 443 | 425 | 461 | -3 | -21 | 15 |  | 320 | 318 | 303 | 333 | 2 | -13 | 17 |
| **Denmark** | 486 | 488 | 481 | 496 | -2 | -10 | 5 |  | 390 | 393 | 387 | 399 | -3 | -9 | 4 |
| **England & Wales** | 567 | 499 | 491 | 508 | 68↑ | 59 | 77 |  | 461 | 411 | 403 | 419 | 50↑ | 41 | 58 |
| **Estonia** | 748 | 720 | 701 | 740 | 28↑ | 9 | 47 |  | 426 | 414 | 403 | 425 | 13↑ | 1 | 24 |
| **France** | 516 | 488 | 482 | 494 | 28↑ | 22 | 34 |  | 361 | 347 | 342 | 352 | 14↑ | 8 | 19 |
| **Georgia** | 1100 | 993 | 972 | 1015 | 107↑ | 85 | 128 |  | 632 | 579 | 564 | 594 | 53↑ | 38 | 69 |
| **Israel** | 444 | 413 | 406 | 421 | 30↑ | 23 | 37 |  | 336 | 326 | 320 | 333 | 9↑ | 3 | 15 |
| **Italy** | 528 | 473 | 465 | 480 | 55↑ | 47 | 62 |  | 423 | 389 | 381 | 397 | 34↑ | 27 | 42 |
| **Mauritius** | 801 | 805 | 780 | 829 | -4 | -28 | 21 |  | 505 | 549 | 531 | 566 | -44↓ | -62 | -26 |
| **Norway** | 417 | 399 | 392 | 406 | 17↑ | 10 | 24 |  | 366 | 370 | 364 | 377 | -4 | -11 | 3 |
| **Peru** | 772 | 409 | 402 | 416 | 363↑ | 356 | 369 |  | 475 | 328 | 322 | 334 | 147↑ | 141 | 153 |
| **Slovenia** | 594 | 529 | 515 | 545 | 65↑ | 50 | 80 |  | 458 | 404 | 394 | 415 | 54↑ | 43 | 64 |
| **Sweden** | 420 | 374 | 369 | 380 | 45↑ | 40 | 51 |  | 348 | 324 | 319 | 330 | 23↑ | 18 | 28 |
| **Ukraine** | 1219 | 1040 | 940 | 1144 | 179↑ | 75 | 279 |  | 531 | 516 | 460 | 574 | 15 | -43 | 71 |
| **USA** | 744 | 634 | 627 | 641 | 110↑ | 103 | 117 |  | 548 | 477 | 471 | 483 | 71↑ | 65 | 77 |

Northern Ireland is excluded due to the lack of sex-specific all-cause mortality data; Scotland is not included due to inability to calculate ASMR.

*For all countries, the sum of observed and expected deaths is up to week 52, with the exception of England & Wales and Scotland (up to week 51), and Mauritius for weeks 2-52

^↑^ Indicates statistically significant excess all-cause mortality using the sum of deaths for the whole of 2020

^↓^ Indicates a statistically significant reduction all-cause mortality using the sum of deaths for the whole of 2020

**Table S7 – Comparison of the cumulative observed and expected mortality rate for the whole year (2020); by age group**

| **Country** | **Observed mortality rate / 100,000 population*** | **Expected mortality rate / 100,000 population *** | **Lower limit of 95% CI of Expected mortality rate** | **Upper limit of 95% CI of mortality rate** | **Difference (Observed-Expected mortality rate)** | **Difference using the Lower limit of 95% CI of Expected mortality rate** | **Difference using the Upper limit of 95% CI of Expected mortality rate** |  | **Observed mortality rate / 100,000 population*** | **Expected mortality rate / 100,000 population *** | **Lower limit of 95% CI of Expected mortality rate** | **Upper limit of 95% CI of mortality rate** | **Difference (Observed-Expected mortality rate)** | **Difference using the Lower limit of 95% CI of Expected mortality rate** | **Difference using the Upper limit of 95% CI of Expected mortality rate** |
| --- | --- | --- | --- | --- | --- | --- | --- | --- | --- | --- | --- | --- | --- | --- | --- |
| **<65** | | | | | | | |  | **65+** | | | | | | |
| **Australia** | 82 | 86 | 84 | 89 | -4↓ | -7 | -1 |  | 2970 | 3042 | 3011 | 3074 | -72↓ | -104 | -41 |
| **Austria** | 176 | 170 | 166 | 174 | 6↑ | 3 | 10 |  | 4564 | 4193 | 4106 | 4281 | 371↑ | 283 | 458 |
| **Brazil** | 306 | 274 | 269 | 278 | 33↑ | 28 | 37 |  | 4563 | 4085 | 4052 | 4119 | 477↑ | 443 | 511 |
| **Denmark** | 164 | 166 | 161 | 170 | -1 | -6 | 3 |  | 3961 | 3993 | 3939 | 4046 | -32 | -85 | 22 |
| **England & Wales** | 187 | 170 | 167 | 174 | 16↑ | 13 | 20 |  | 4612 | 4033 | 3949 | 4119 | 579↑ | 493 | 663 |
| **Georgia** | 397 | 366 | 357 | 374 | 32↑ | 23 | 40 |  | 6648 | 6001 | 5850 | 6154 | 647↑ | 495 | 798 |
| **Israel** | 103 | 103 | 100 | 106 | 0 | -3 | 3 |  | 3557 | 3321 | 3269 | 3373 | 236↑ | 184 | 287 |
| **Italy** | 156 | 149 | 146 | 153 | 7↑ | 3 | 10 |  | 4617 | 3125 | 2922 | 3333 | 1492↑ | 1284 | 1695 |
| **N. Ireland** | 197 | 183 | 175 | 191 | 14↑ | 5 | 22 |  | 4486 | 4043 | 3918 | 4169 | 443↑ | 318 | 568 |
| **Norway** | 122 | 123 | 119 | 126 | -1 | -4 | 3 |  | 3692 | 3707 | 3652 | 3762 | -15 | -70 | 40 |
| **Peru** | 255 | 154 | 150 | 157 | 101↑ | 98 | 105 |  | 4659 | 2763 | 2719 | 2808 | 1896↑ | 1851 | 1940 |
| **Slovenia** | 203 | 198 | 191 | 206 | 5 | -3 | 12 |  | 4815 | 4130 | 4031 | 4228 | 685↑ | 587 | 784 |
| **USA** | 317 | 270 | 265 | 275 | 47↑ | 42 | 51 |  | 4533 | 3918 | 3888 | 3947 | 615↑ | 585 | 645 |
| **<70** | | | | | | | |  | **70+** | | | | | | |
| **Brazil** | 271 | 241 | 237 | 246 | 29↑ | 25 | 34 |  | 2935 | 2570 | 2547 | 2592 | 365↑ | 343 | 387 |
| **Cyprus** | 173 | 173 | 163 | 183 | 1 | -9 | 11 |  | 4830 | 4855 | 4679 | 5032 | -25 | -202 | 151 |
| **Denmark** | 160 | 162 | 158 | 166 | -2 | -6 | 2 |  | 4898 | 4901 | 4829 | 4974 | -3 | -75 | 70 |
| **Estonia** | 390 | 365 | 353 | 377 | 25↑ | 13 | 37 |  | 5941 | 5912 | 5772 | 6052 | 30 | -111 | 169 |
| **France** | 242 | 241 | 236 | 245 | 1 | -3 | 5 |  | 5184 | 4801 | 4722 | 4879 | 383↑ | 304 | 461 |
| **Georgia** | 529 | 482 | 472 | 492 | 47↑ | 37 | 57 |  | 8802 | 7985 | 7779 | 8193 | 817↑ | 609 | 1023 |
| **Italy** | 219 | 204 | 200 | 208 | 14↑ | 10 | 18 |  | 5813 | 5180 | 5078 | 5283 | 633↑ | 530 | 735 |
| **Mauritius** | 484 | 504 | 490 | 519 | -20↓ | -35 | -6 |  | 5558 | 5825 | 5624 | 6030 | -268↓ | -472 | -66 |
| **Norway** | 171 | 170 | 166 | 174 | 1 | -3 | 5 |  | 4804 | 4823 | 4747 | 4898 | -18 | -94 | 57 |
| **Sweden** | 167 | 156 | 153 | 159 | 11↑ | 8 | 15 |  | 5106 | 4600 | 4534 | 4666 | 506↑ | 440 | 572 |
| **Ukraine** | 492 | 551 | 509 | 594 | -59↓ | -103 | -17 |  | 8744 | 7274 | 6687 | 7877 | 1470↑ | 867 | 2057 |

Scotland is not included due to unavailability of age-specific weekly mortality data.

*For all countries, the sum of observed and expected deaths is up to week 52, with the exception of England & Wales and Scotland (up to week 51), N. Ireland (up to week 50), and Mauritius for weeks 2-52

^↑^ Indicates statistically significant excess all-cause mortality using the sum of deaths for the whole of 2020

^↓^ Indicates a statistically significant reduction all-cause mortality using the sum of deaths for the whole of 2020

**Figure S1 Weekly z-score of crude all-cause mortality rate for total population**

**
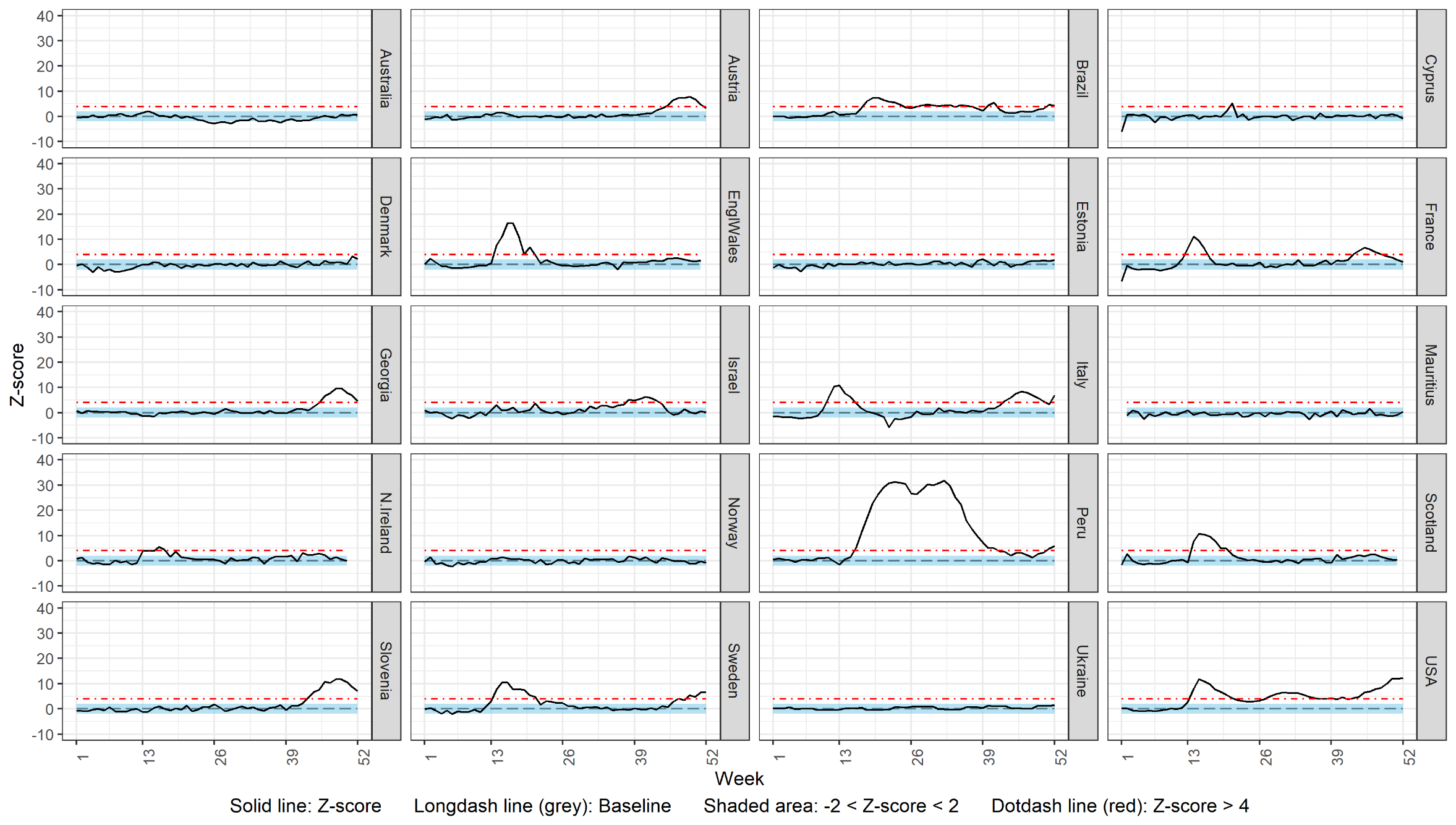
**

**Figure S2 – Weekly z-score of crude all-cause mortality rate by sex**

**
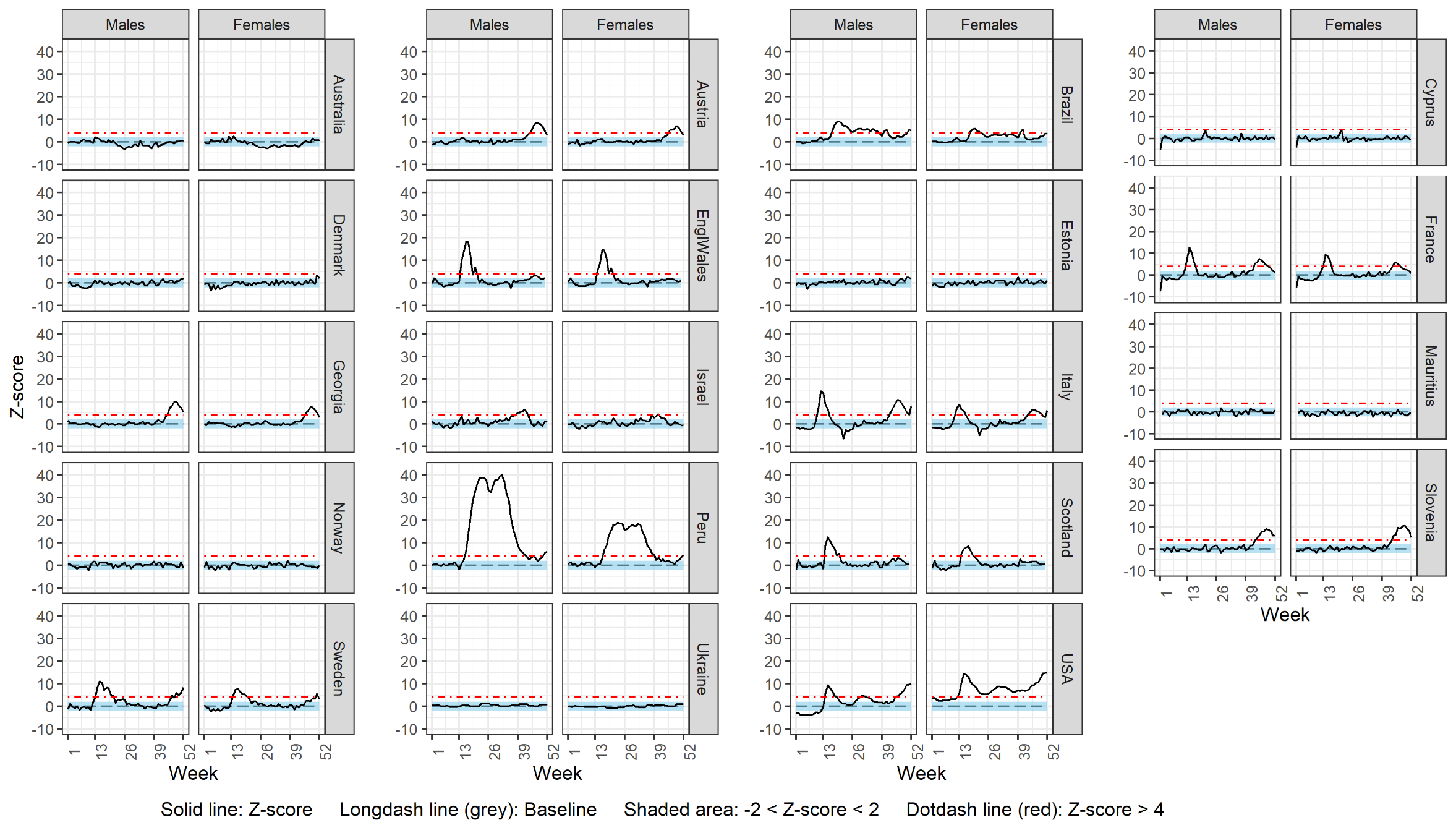
**

**Figure S3 – Observed against expected mortality rate by more detailed age breakdowns for the countries observing substantial excess mortality in younger age groups**


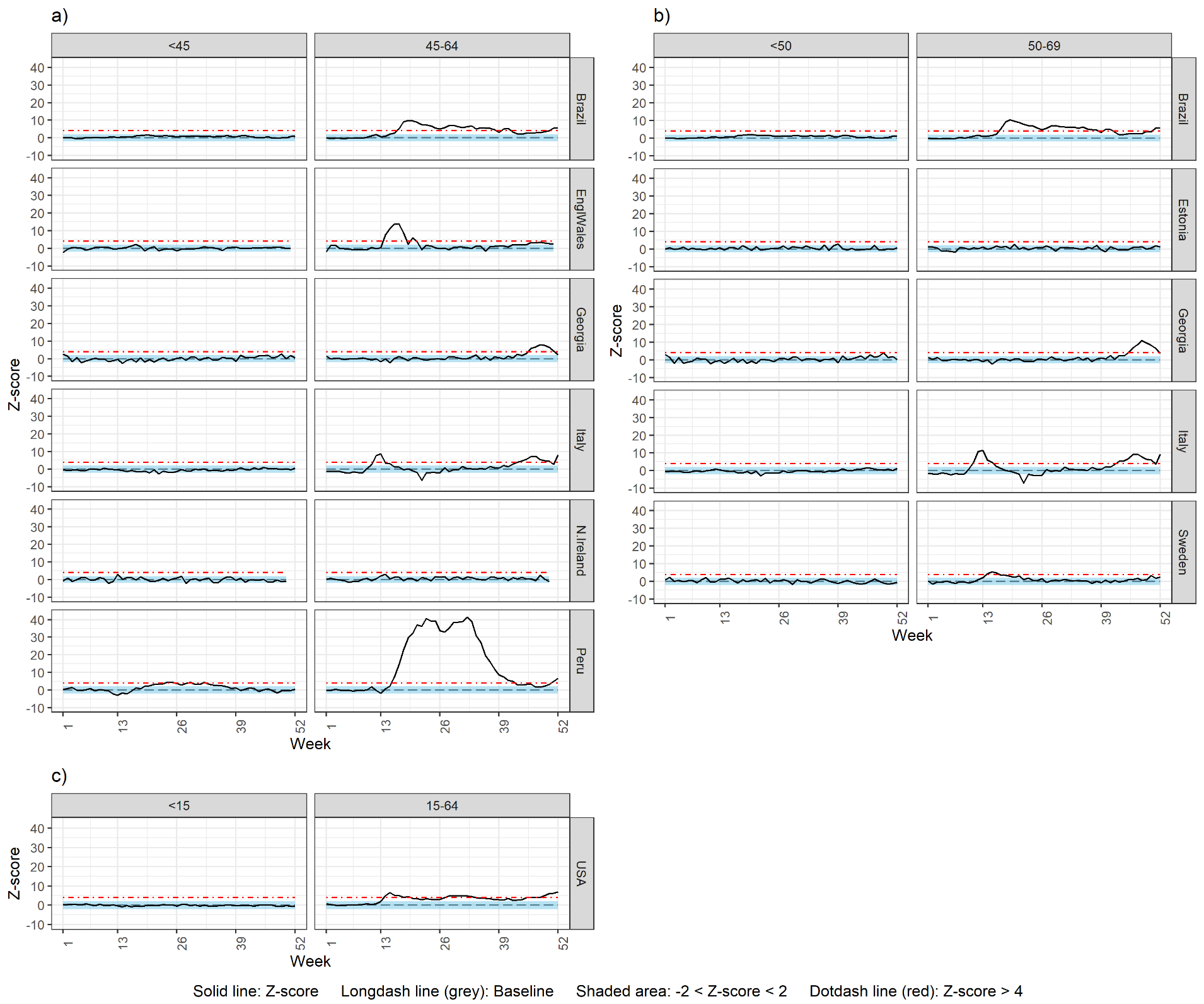

Supplement: dyac170_Supplementary_Data [file dyac170_supplementary_data.docx]
